# Effects of Infant Massage: A Systematic Review

**DOI:** 10.3390/ijerph19116378

**Published:** 2022-05-24

**Authors:** Rebecca Mrljak, Ann Arnsteg Danielsson, Gerth Hedov, Pernilla Garmy

**Affiliations:** 1 Faculty of Health Sciences, Kristianstad University, SE-291 88 Kristianstad, Sweden; rebeccamrljak@gmail.com (R.M.); annmonica73@hotmail.com (A.A.D.); gerth.hedov@hkr.se (G.H.); 2 Faculty of Medicine, Lund University, SE-221 85 Lund, Sweden

**Keywords:** infant massage, child health care, review, intervention, infant

## Abstract

Infant massage is performed in various international contexts. There is a need for an updated literature review on this topic. The purpose of the current review was to investigate the effects of infant massage. A systematic literature review was conducted to investigate the effects of infant massage on the following outcomes: pain relief, jaundice, and weight gain. The inclusion criteria were infants from 0–12 months. The literature search was performed until January 2022, using the CINAHL, PubMed, and PsycINFO databases, and included studies published from 2017–2021, returning 16 RCT/CCT studies with a total of 1416 participating infants. A review template was used by two independent reviewers to assess the risk of bias in the included studies. The results were synthesized and presented in the form of tables and narratives. In five of seven studies (*n* = 422 resp. *n* = 717) investigating pain relief, infant massage was found to alleviate pain. In all six studies (*n* = 455) investigating effects on infant massage and jaundice, beneficial effects were found on bilirubin levels. In all four studies (*n* = 244) investigating weight gain, increased weight gain was found among participants who received infant massage. The present literature review provides an indication of the current state of knowledge about infant massage and identifies its positive effects; however, the results must be interpreted with caution. Infant massage may be effective at relieving pain, improving jaundice, and increasing weight gain. Although statistically significant differences were not found between all experimental and control groups, no adverse effects of infant massage were observed. By placing the aforementioned effects in the context of child health care, infant massage may prove beneficial on these outcomes. Given the dearth of research on infant massage in the context of child health care, further research is warranted.

## 1. Introduction

Infant massage is described as a structured touch of the skin, and in many cultures, it is a tradition that begins immediately after birth. Performing infant massage differs worldwide with respect to duration, intensity, extent, use of oil, and parental involvement [1]. Neonatal intensive care units are typically stressful environments for newborns that are largely devoid of human touch. Infant massage has been used in neonatal intensive care units to some benefit for various outcomes such as weight gain, reduced length of stay at hospital and postnatal complications [2].

Infants are defined as a newborn child between the ages of 0–12 months. During infancy, children develop rapidly [3]. According to the American psychoanalyst John Bowlby, attachment begins immediately at birth [4]. Children and mothers interact; the mother reacts to the child’s signals and adapts her behaviors accordingly, and over time the child develops the ability to respond to its mother’s behaviors. It has been shown that mothers who learned and performed infant massage during a hospital stay experienced reduced anxiety and a stronger attachment with their child. Improved attachment was also seen in studies by Guröl [5] and Holditch-Davis et al. [6]. Furthermore, the incidence of depression was reported as 12% in mothers [7] and 6% in fathers [7,8] in the postnatal period. Because postpartum depression is the most common postpartum complication, new mothers are offered screening with the Edinburgh Postnatal Depression Scale (EPDS) six to eight weeks after delivery in Swedish child health care [9]. Depression in new mothers can also negatively affect the father, the child, and parent–child attachment [9]. According to Bowlby’s [4] theory, good parent–child attachment is important and entails that the child feels safe, yielding better conditions for exploration and development [4]. Infant massage improved mothers’ mental well-being [10] and reduced anxiety, depression, and stress [11]. Mothers who continued to massage their children post-discharge showed sustained reductions in anxiety and stress [6]. These effects were not limited to mothers and extending also to fathers, who reported experiencing less stress when attending infant massage courses [12]. These effects could be attributed to the hormone oxytocin, which is secreted as a result of physical contact [13]. Both mothers and children had increased levels of oxytocin during the infant massage. Earlier literature reviews have found some evidence of the effect of infant massage on pain relief [14], reduction of jaundice [15], and weight gain [16]; however, an updated literature review is warranted. The purpose of the study was to review the effects of infant massage.

## 2. Materials and Methods

### 2.1. Design

To determine the current state of knowledge about infant massage, a systematic literature review was performed according to the PRISMA guidelines [17]. A systematic literature review compiles existing research in a specific area in a predetermined way and can form the basis for evidence-based health care.

### 2.2. Study Selection

The international concept of PICO (Population, Intervention, Control, Outcome) was used in this literature review and is defined as follows:

- Population: infants from 0–12 months
- Intervention: infant massage administered by parents or professionals
- Control group: care as usual or other intervention
- Outcome: pain relief, jaundice, weight gain

Because data from controlled studies are considered the most robust [18], only articles that used randomized controlled trials (RCTs) and clinically controlled trials (CCTs) were included in the sample. Additional inclusion criteria consisted of peer-reviewed articles written in English and published between 2017 and 2021. 

### 2.3. Data Collection

The databases CINAHL, PubMed, and PsycINFO were searched, and the studies that fulfilled the inclusion criteria were selected. In order to obtain scientific articles for a chosen purpose, subject words must be searched for individually and in search blocks. Three search blocks—infant, massage, and method—were used. For each search block, subject words and free text were combined with the Boolean term “OR”. The three search blocks were then combined with the Boolean term “AND”. 

Online searches were performed up to January 2022. These searches yielded a total of 122 articles among the three databases; 35 articles in CINAHL, 78 articles in PubMed, and nine articles in PsycINFO. Some of these articles (*n* = 10) were duplicates (i.e., the same article appearing in more than one database).

**Figure 1:**
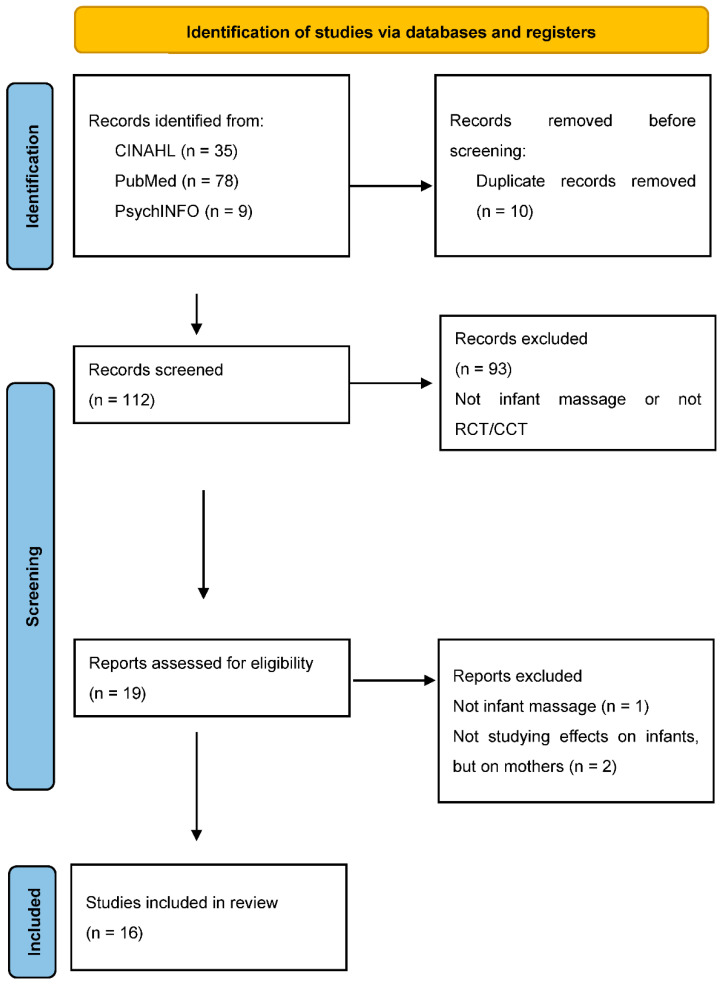
PRISMA flow diagram of the study selection process. *From:* Page, M.J.; McKenzie, J.E.; Bossuyt, P.M.; Boutron, I.; Hoffmann, T.C.; Mulrow, C.D.; Shamseer, L.; Tetzlaff, J.M.; Akl, E.A.; Brennan, S.E.; et al. *The PRISMA 2020 statement: an updated guideline for reporting systematic reviews*. *BMJ* **2021**, *372*, n71. doi: 10.1136/bmj.n71 [17]. For more information, visit: http://www.prisma-statement.org/ (accessed on 1 April 2022).

After the duplicates were removed, the title and abstract of 112 articles were reviewed. Articles whose title and abstract did not meet the inclusion criteria were excluded (e.g., the article focused on another type of massage, such as cardiac massage, massage on the mother, or reflexology; review articles; or articles that did not examine the outcome in PICO). After this initial review of the title and abstract, a total of 19 articles were reviewed in their entirety; however, one article was excluded since it did not examine infant massage, and two articles were excluded since they did not examine the outcome in PICO (Figure 1). 

### 2.4. Method of Analysis

The 16 articles were read in their entirety and independently critically reviewed several times according to the review template. After individual review of the first five articles, the first and second author reviewed them together to ensure that the review template was being applied in a similar way and to discuss any potential changes to the template. An amendment was added to the review template to describe potential bias from blinding. The review then continued independently and jointly thereafter (Figure 1). All articles included in this review are presented in Appendix A. 

## 3. Results

Table 1 shows the total number of participants within each subject category and the number of participants in the experimental group among the studies whose results were statistically significant. One study [19] examined the effects of infant massage on both weight gain and pain relief.

### 3.1. Study Characteristics

The studies included 15 RCTs and one CCT, and the study quality was judged to be medium (*n* = 9) or high (*n* = 7). The studies were conducted in the following countries: Iran (*n* = 7), Turkey (*n* = 4), China (*n* = 2), India (*n* = 1), Taiwan (*n* = 1) and USA (*n* = 1).

### 3.2. Pain Relief

The effects on pain were examined in seven studies that included a total of 717 children [20,21,22,23,24,25,26]. Pain during blood sampling was examined in five studies [20,22,24,25,26], postoperative pain was examined in one study [21], and colic pain was examined in one study [23]. More than half of the studies showed a significant difference in pain between the intervention and control group, regardless of the measuring instrument, type of massage, and the child’s gestational and actual age [20,22,25,26].

#### 3.2.1. Pain during Blood Sampling

In the five studies that examined pain during sampling, pain during massage was reduced compared to controls [20,22,24,25,26]; in four of these studies, the difference was statistically significant [20,22,25,26]. In the study by Roshanray et al. [24], two interventions (mother’s hug and infant massage) were compared against the control group. Although some non-significant improvement was found in the massage group, the mother’s hug was found to have a greater impact on pain compared to both the control and massage groups [24]. Zargham-Boroujeni et al. [26] examined the pain relieving effects of infant massage compared to breastfeeding and controls, finding that massage relieved pain more than did breastfeeding.

The gestational age of the children in the studies varied, with both premature and full-term children being examined. In three of the studies, healthy full-term children (*n* = 402) born at weeks 37–42 were examined [22,24,25]. Zargham-Boroujeni et al. [26] examined full-term or near-full-term children (*n* = 75), where gestational age was described as older than 34 weeks, whereas Chik et al. [20] examined children (*n* = 80) born between weeks 30–40. The children in all studies [20,22,24,25,26] were described as newborns, and Chik et al. [20] clarified that the children were up to one week old.

To measure pain, the Neonatal Infant Pain Scale (NIPS) was used in four studies [22,24,25,26]; the fifth study used the Premature Infant Pain Profile (PIPP) [20].

The children were massaged for a relatively brief period (2–3 min) prior to test administration, and the type of massage varied. Foot massage was described in three studies [22,24,25], massage at the sampling site in one study [26], and massage of the upper extremities in one study [20]. The massage was administered by different individuals, including researchers [22,24,26], mothers [25], and nurses [20].

The sampling method differed among the five studies. Heel stick sampling was used in two studies [22,25], venous sampling was used in two studies—although the location was unclear—[20,26], and blood sampling was used in the fifth study, with unclear localization [24]. Among the studies, the follow-up period varied between 30 s and 5 min [20,22,24,25,26].

#### 3.2.2. Postoperative Pain

Harrison et al. [21] examined postoperative pain in children (aged 1–12 months) with heart disease surgery with or without full body massage performed for 30 min once daily by a massage therapist. The pain was measured with the Face, Legs, Activity, Cry, Consolability (FLACC) pain assessment tool, and the follow-up period was 1 week [21]. Compared to patients who received daily rest, the pain estimates for patients who received the treatment were lower on each treatment day, except for the last (day seven); however, this difference was not statistically significant.

#### 3.2.3. Colic Pain

The effect of infant massage on colic pain in children under 12 weeks of age was investigated by Nahidi et al. [23]. The children’s crying behavior improved significantly from the first to the last day of intervention within both the massage group and the group that was rocked. The sleep pattern improved significantly within the groups, which was interpreted as reduced pain. Full body massage was performed for 15–20 min twice daily by the mother. The pain was measured by the mother documenting different crying behaviors, sleep duration, and performing a pain assessment, all using the McGill Pain Scale, with a follow-up period of one week [23].

### 3.3. Jaundice

Bilirubin levels were measured in six of the included studies to evaluate the effect of infant massage on reducing jaundice [19,27,28,29,30,31]. The six studies examined a total of 455 children; in five of these studies, children with jaundice were examined and treated with phototherapy [19,27,29,30,31]. However, Gözen et al. [28], assessed healthy children to determine whether and to what extent they developed jaundice with or without massage. In the studies examining children with jaundice, a significant difference in serum bilirubin levels was found after massage [19,27,29,30,31]. Some relate this to an increase in the frequency of bowel movements [27,30,31]. Jazayeri et al. [29] compared the effects of massage and reflexology. Both massage and reflexology were found to yield significant improvements in bilirubin levels; however, no significant difference was found between reflexology and massage [29]. Kenari et al. [31] found that the kangaroo method and the massage significantly reduced bilirubin levels, with no significant differences between these two interventions. In the only study on healthy children, Gözen et al. [28] found that children who received massage had a significantly lower transcutaneous bilirubin increase. All studies showed a significant decrease in bilirubin levels between the intervention and control groups, regardless of the measuring instrument, type of massage, and the children’s gestational and actual ages [19,27,28,29,30,31].

Premature and full-term children were examined in the six studies. Four of the studies examined full-term infants (*n* = 374) born at weeks 37–42 [27,28,30,31]. Jazayeri et al. [29] examined children (*n* = 51) whose gestational age was higher than 35 weeks, whereas Rimpy [19] examined children (*n* = 40) who were premature or full-term. All studies described the children as newborns [19,27,28,29,30,31]. In some of the studies, the age of the children was further specified: Eghbalian et al. [27] examined children who were 1–14 days old; Lori Kenari et al. [31] examined children older than 48 h; and Rimpy [19] examined children who were one day to one month old.

Bilirubin was measured by venous sampling in four of the studies [27,29,30,31] and by measuring transcutaneous bilirubin in one study [28]. Another study failed to specify how bilirubin was measured [19].

Different types of massage were performed among the six studies. Mainly, full-body massage had a duration of 5–15 min per session, with two to three sessions per day [19,27,29,30,31]. One study only performed abdominal massage for a duration of 5 min per session, with three sessions per day [28]. The follow-up period varied among studies, and the children received the massage regimen over a period of two to four days [19,27,28,31]. Two of the studies failed to specify the duration of the follow-up period [29,30], one of which explains that the massage was given during phototherapy treatment [30]. Among the studies, the massage was performed by either the researcher [19,30,31], the nurse [28], or the mother [27]. One study failed to specify who performed the massage [29].

### 3.4. Weight Gain

Four of the included studies examined the effects of infant massage on weight gain in a total of 244 children [19,32,33,34]. A significant weight gain following regular massage was shown in all four studies [19,32,33,34]. Liao et al. [32] reported that massage with medium-chain triglycerides (MCT) oil had a significantly better effect of weight gain on days five to seven of massage compared to both the group that received massage without oil and the control group. In two of the studies, 10 mL/kg of oil were used per day during the massage [32,33]. All studies showed a significant difference in weight gain between the intervention and control group, regardless of measuring instrument, type of massage, and the child’s gestational and actual age [19,32,33,34].

The gestational age of the children in the studies varied. In three of the studies, premature babies (*n* = 204) born at weeks 28–37 [32], weeks 30–36 [33], and weeks 30–34 [34] were examined, but their actual ages were not stated. In the study by Rimpy [19], children who were premature or full-term (*n* = 40) and aged one day to one month old were examined.

In four studies [19,32,33,34], the weight of the children was measured. In the study by Liao et al. [32], the children were weighed by two experienced nurses, using a digital scale that was regularly calibrated. Zhang and Wang [34] measured weight with a standardized instrument, and the same nurse performed and recorded all measurements. In the studies of Rimpy [19] and Taheri et al. [33], no description was provided of measuring instruments or who performed the measurements. Among these four studies [19,32,33,34], different types of full body massage were performed for 10–15 min per session, with two to three sessions per day for a total period ranging from three days to two weeks. The massage was performed either by the mother [34], nurses [32], or the researcher [19]; the study by Taheri et al. [33] failed to clarify whether the researcher or nurse performed the massage.

## 4. Discussion

The literature review presents 16 clinical studies—all of which are RCTs or CCTs—published between 2017–2021, that report the effects of infant massage. Based on our review, there appear to be positive effects of infant massage on pain relief, jaundice, and weight gain. The most distinctive findings are discussed below and related to Bowlby’s [4] attachment theory.

Infant massage can have pain-relieving effects in various conditions. Thus, infant massage may be relevant to child health care professionals in the context of painful procedures and in the treatment of other types of pain in infants. Infant massage relieved pain when performed immediately before sampling venously or by heel stick. Even in colic, infant massage had beneficial effects on pain. Garmy [10] found that the evidence for the analgesic effects of infant massage was unclear. In contrast, Jain et al. [35] found that pain after heel stick was improved when infant massage was performed before sampling. Pillai Riddell et al. [36] confirmed that infant massage in conjunction with painful procedures, such as heel stick sampling, injections, and venipuncture could alleviate pain in infants. That infant massage reduced the duration of crying in children suffering from colic was also reported by Çetinkaya and Başbakkal [37]. This finding is corroborated by other studies reporting that infant massage is an effective treatment for colic [37,38,39].

The infant massage’s pain-relieving effects can be applied to vaccination of infants in child health care. This pain-relieving effect is supported by Esfahani et al. [40], who found that infant massage had a pain-relieving effect during injections. Within child health care, various forms of pain relief are recommended for vaccination of children, such as sugar solution, local anesthesia, breastfeeding, injection techniques, and distraction. These methods can also be used in combination to optimize results. Breastfeeding was even more effective at alleviating pain than was infant massage while receiving an injection [40], which may explain why breastfeeding is recommended as pain relief during injections. However, the evidence also suggests that the effects of infant massage on pain during sampling can be positive. 

Only one study in the review indicated pain-relieving effects of infant massage on colic [23]. The study was deemed to be of medium quality and used a measuring instrument that was not validated for use in children [23]. Although the result should be interpreted with caution, it may nonetheless be relevant in the pediatric health care context, in which infantile colic is relatively common. For parents, the condition can be extremely stressful and impart consequences for mental health [41]. A vicious circle can arise in which parents’ interaction with and ability to comfort their child is affected by stress. Postnatal depression may also occur, which can, in turn, lead to profound consequences for the child [41]. A deteriorating interaction has an effect on the attachment between parent and child, according to Bowlby’s [4] theory. To reduce colic, infant massage is recommended [37]. In this context, child health care professionals have important roles, because teaching infant massage as a pain-relieving treatment option for colic may positively affect the parent–child attachment. 

Infant massage can counteract jaundice in newborns. Children with jaundice showed improved bilirubin levels as a result of infant massage combined with conventional phototherapy. Infant massage also acted prophylactically against jaundice in healthy newborns. An increase in the frequency of bowel movements was considered to be an underlying cause of a decrease in bilirubin levels. Also, meta-analyses by Abdellatif et al. [42] and Lei et al. [15] showed that infant massage was an effective treatment for jaundice. High bilirubin levels were found in newborn meconium, which could be absorbed into the blood [15]. As a result of infant massage, the frequency of bowel movements increased and, thus, bilirubin levels decreased [42].

Infant massage can have beneficial effects for weight gain in premature babies, and massage can be applied in all children. Full body massage had a positive effect on premature infants’ weight gain. The positive effect of infant massage on weight gain in premature infants has also been demonstrated in other studies [2,43,44]. Additional studies also report the beneficial effects of infant massage on weight gain in non-premature infants [1,2,10,43,45].

### Strengths and Limitations

A strength of this review was the rigorous double screening of titles, abstracts, and full texts, which mitigated the risk of systematic bias at the screening stages whilst also decreasing the total number of errors or missed studies.

Limitations of this review were that it only included studies published during the last five years and studies published in English and in the databases CINAHL, PubMed, and PsycINFO, thereby excluding potentially relevant studies in other languages or databases.

## 5. Conclusions

The present literature review provides an indication of the current state of knowledge about infant massage and identifies its positive effects, although the results must be interpreted cautiously. Infant massage may have tendencies to relieve pain, improve jaundice and increase weight gain. Although statistically significant differences were not found between all experimental and control groups, no adverse effects of infant massage were observed. By placing the above-mentioned effects in the context of child health care, the use for infant massage can be developed. Given the dearth of research on infant massage in the child health care context, more research is warranted. 

## Figures and Tables

**Table 1:** Effect of infant massage on pain relief, jaundice and weight gain.

Outcome	Studies Published in 2017–2021 (*n* = 16)	Total Participants (*n*)/Participants with Stastistically Significant Results (*n*)
Pain relief	7	717/422
Jaundice	6	455/455
Weight gain	4	244/244
**Total**		**1416/1121**

## Registration and Protocol

A study protocol including a review template was registered in March 2021 at Kristianstad University. It is publicly available upon request.

## Appendix A. Included Studies

**Table A1:** Included studies.

Author, Year, Country	Design	Inclusion Criteria	Intervention and Control Groups	Number of Participants/Drop-outs	Time fo Follow-Up	Effects of Infant Massage	Study Quality	Outcome
Chik et al., 2017 China	RCT	Premature and full-term babies (weeks 30–40)	I: massage C: care as usual (cross-over)	I: 40 C: 40 Drop-out: 15	30 s	Less pain at venous sampling *	High	Pain relief
Eghbalian et al., 2017 Iran	RCT	Full-term babies with jaundice	I: massage with baby oil and phototherapy C: phototherapy	I: 67 C: 67 Drop-out: –	4 days	Decreased levels of bilirubin day 3 and 4 *	Medium	Jaundice
Gözen et al., 2019 Turkey	RCT	Healthy, full-term babies	I: massage with baby oil C: care as usual	I: 44 C: 46 Drop-out: 0	48 h	Lower transcutan bilirubin increase *	High	Jaundice
Harrison et al., 2020 USA	RCT	Babies (1 day–12 months) with CCHD (Complex Congenital Heart Disease) who received their first thorax surgery	I: massage with body lotion C: rest	I: 30 C: 30 Drop out: 0	7 days	Less pain all days but day 7. NS	Medium	Pain relief
Jazayeri et al., 2021 Iran	RCT	Babies with jaundice (> week 35)	I (1): reflexology with olive oil and photo therapy I (2): massage and phototherapy C: phototherapy	I (1): 17 I (2): 17 C: 17 Drop-out: 5	Not presented	Decreased levels of bilirubin in I (1) and I (2). *	Medium	Jaundice
Korkmaz & Esenay, 2020 Turkey	RCT	Full-term babies with jaundice	I: massage with baby oil and phototherapy C: phototherapy	I: 25 C: 25 Drop-out: 30	During ongoing photo therapy	Less total serum bilirubin *	High	Jaundice
Liao et al., 2021 Taiwan	RCT	Prematur babies (week 28–37)	I (1): massage with MCT oil I (2): massage without oil C: care as usual	I (1): 16 I (2): 16 C: 16 Drop-out: 3	7 days	Day 5–7 greater weight gain in I (1) compared with I (2) and C *	High	Weight gain
Lori Kenari et al., 2020 Iran	RCT	Full-term babies with jaundice	I (1): massage with sunflower oil and photo therapy I (2): the kangaroo method and phototherapy C: phototherapy	I (1): 30 I (2): 30 C: 30 Drop-out: -	72 h	Less bilirubin leves in I (1) och I (2) *	Medium	Jaundice
Nahidi et al., 2017 Iran	RCT	Babies with colic	I: massage with baby oil C: rocking	I: 50 C: 50 Drop-out: –	1 week	Decreased crying incidents, crying duration and crying intensity in both I and C *	Medium	Pain relief
Özkan et al., 2019 Turkey	RCT	Healthy full-term babies	I (1): acupressure I (2): massage C: care as usual	I (1): 46 I (2): 47 C: 46 Drop-out: 11	1 min	Less pain during and 1 min after samping in I (1) och I (2). *	Medium	Pain relief
Rimpy & Singh, 2018 India	CCT	Premature and full-term babies with jaundice	I: massage with almond oil and phototherapy C: phototherapy	I: 20 C: 20 Drop-out: –	3 days	Weight gain * Decreased levels of bilirubin *	Medium	Jaundice/ Weight gain
Roshanray et al., 2020 Iran	RCT	Healthy full-term babies	I (1): mother’s hug I (2): massage C: care as usual	I (1): 45 I (2): 45 C: 45 Drop-out: 15	5 min	Shorter crying duration in I (1) och I (2) compared with C *	High	Pain relief
Taheri et al., 2018 Iran	RCT	Healthy premature babies (week 30–36)	I: massage with sunflower oil C: care as usual	I: 22 C: 22 Drop-out: 0	5 days	Improved weight gain *	High	Weight gain
Yavaş et al., 2021 Turkey	RCT	Healthy full-term babies	I: foot massage C: care as usual	I: 64 C: 64 Drop-out: 0	3 min	Less pain during and after sampling *	High	Pain relief
Zargham-Boroujeni et al., 2017 Iran	RCT	Full-term or almost full-term babies (> week 34)	I (1): breastfeeding I (2): massage C: care as usual	I (1): 25 I (2): 25 C: 25 Drop-out: –	30 s	Reduced pain in I (1) and I (2) * More pain relief in I (2) compared with I (1). *	Medium	Pain relief
Zhang & Wang, 2019 China	RCT	Prematur babies (week 32–34)	I: massage C: care as usual	I: 54 C: 58 Drop-out: –	2 weeks	Improved weight gain *	Medium	Weight gain

* = statistically significant difference (*p* < 0.05). I = intervention. C = control. NS = non-significant difference.
